# Alarming Hemorrhage

**Published:** 1869-10

**Authors:** H. Scott


### ALARMING HEMORRHAGE.

H. SCOTT.

A week since, a young man called at sundown to have the second lower molar on the right side removed. He was of sanguine temperament, florid complexion, in good health, twenty-two years of age, and had worked through the day at his trade of machinist. The ensuing hemorrhage was ordinary and he went away. At eleven o'clock he called me up. He had gone to sleep, and waked in an hour deluged with blood. I found his pulse 100, bounding and quick. The sockets were firmly packed with cotton saturated with sulphate of iron, which very partially retained the jets of arterial blood. An hour passed, and I was thinking of employing constitutional treatment, when a syncope brought my patient to the floor. The pulse fell to 70 ; perspiration broke out profusely ; the flow of blood ceased ; and the case was ended. He resumed work in two days, and is all right. Yesterday he had the corresponding tooth extracted, with nothing unusual following.

